# *Spirulina maxima* Extract Prevents Neurotoxicity via Promoting Activation of BDNF/CREB Signaling Pathways in Neuronal Cells and Mice

**DOI:** 10.3390/molecules22081363

**Published:** 2017-08-17

**Authors:** Eun-Jeong Koh, Young-Jin Seo, Jia Choi, Hyeon Yong Lee, Do-Hyung Kang, Kui-Jin Kim, Boo-Yong Lee

**Affiliations:** 1 Department of Food Science and Biotechnology, College of Life Science, CHA University, Seongnam, Kyonggi 13488, Korea; kej763@naver.com (E.-J.K.); yj2477@hanmail.net (Y.-J.S.); wldk3176@gmail.com (J.C.); 2 Department of Food Science and Engineering, Seowon University, Cheongju 28674, Korea; hyeonl@kangwon.ac.kr; 3 Jeju International Marine Science Center for Research & Education, Korea Institute of Ocean Science & Technology (KIOST), Jeju 63349, Korea; dohkang@kiost.ac.kr

**Keywords:** *Spirulina maxima* 70% ethanol extract (SM70EE), trimethyltin (TMT), neurotoxicity, acetylcholinesterase (AChE), cognitive deficits

## Abstract

*Spirulina maxima* is a microalgae which contains flavonoids and other polyphenols. Although *Spirulina maxima* 70% ethanol extract (SM70EE) has diverse beneficial effects, its effects on neurotoxicity have not been fully understood. In this study, we investigated the neuroprotective effects of SM70EE against trimethyltin (TMT)-induced neurotoxicity in HT-22 cells. SM70EE inhibited the cleavage of poly-ADP ribose polymerase (PARP). Besides, ROS production was decreased by down-regulating oxidative stress-associated enzymes. SM70EE increased the factors of brain-derived neurotrophic factor (BDNF)/cyclic AMP-responsive element-binding protein (CREB) signalling pathways. Additionally, acetylcholinesterase (AChE) was suppressed by SM70EE. Furthermore, we investigated whether SM70EE prevents cognitive deficits against scopolamine-induced neurotoxicity in mice by applying behavioral tests. SM70EE increased step-through latency time and decreased the escape latency time. Therefore, our data suggest that SM70EE may prevent TMT neurotoxicity through promoting activation of BDNF/CREB neuroprotective signaling pathways in neuronal cells. In vivo study, SM70EE would prevent cognitive deficits against scopolamine-induced neurotoxicity in mice.

## 1. Introduction

Neurotoxicity induces neuronal death and dysfunction of the neuronal network in neuronal cells [1]. Trimethyltin chloride (C_3_H_9_ClSn, TMT) and scopolamine are known to cause cognitive disorders by inducing neurotoxicity, [2,3,4]. TMT is a chemical compound which causes neuronal damage by inducing neuronal cell apoptosis in the hippocampus, eventually leading to neurotoxicity. Neurotoxicity produced by TMT also involves several molecular mechanisms such as oxidative stress, mitochondrial dysfunction, and neurotransmitter dysfunction [2]. Scopolamine, which acts as an inducer of cognitive deficits [5], has been used to study learning and memory impairment [6,7]. Recently, scopolamine was shown to generate neurotoxicity which increases cholinergic dysfunction and oxidative stress [8,9].

Diverse signaling pathways are related with TMT neurotoxicity. TMT increases the cleavage of poly (ADP-ribose) polymerase (PARP) related with apoptotic processes in neuronal cells [10]. In addition, TMT neurotoxicity augments activation of nicotinamide adenine dinucleotide phosphate hydrogen (NADPH) oxidase4 (NOX4) and reactive oxygen species (ROS) production [11]. The other factor, heme-oxygenase 1 (HO-1) is an enzyme which catalyzes the conversion of heme into biliverdin in response to oxidative stress [12,13]. Recently, a study has demonstrated that nuclear factor erythroid 2-related factor 2 (Nrf2)/HO-1 signaling pathways is increased by TMT and proceeds neuronal cell death [14]. 

Another mechanism of the neuroprotective signaling pathway damage caused by TMT neurotoxicity is activated by the response to TMT-induced neuronal damage. Neuronal cells damaged by TMT neurotoxicity release brain-derived neurotrophic factor (BDNF), which binds to tropomyosin-related kinase receptor type B (TrkB) and then phosphorylates TrkB. The phosphorylation of TrkB expresses the cyclic AMP responsive element binding protein (CREB) and increases BDNF transcription. Consequently, BDNF proceeds to protect neuronal cells from damage and to induce neurogenesis in response to neurotoxicity [2,15]. Additionally, acetylcholine (ACh) mediates neurotransmission for improving memory and learning function in the central nervous system. ACh is hydrolyzed by AChE which is an enzyme that breaks it down into choline and acetate [16]. Recently, a study has revealed that acetylcholinesterase (AChE), which is one of the risk factors of Alzheimer’s disease (AD), is increased by TMT [4,17].

*Spirulina maxima (S. maxima)* is a spiral-shaped microalgae which contains flavonoids and other polyphenols such as C-phycocyanin (C-PC) and chlorophyll a [18,19,20]. Many studies have shown that *S. maxima* has various beneficial effects, such as anti-inflammatory and anti-oxidant actions [20,21]. However, the mechanism behind the neuroprotective effects of *S. maxima* 70% ethanol extract (SM70EE) against neurotoxicity it is not fully known. 

HT-22 cells, a type of mouse hippocampal cell, are commonly used in in vitro studies related with cognitive disorders. Many studies have demonstrated the neuroprotective effects of different phytochemicals on cognitive deficits and neurotoxicity via using HT-22 cells [22,23]. In addition, generally, mice are used to conduct behavior test in vivo studies of cognitive disorders [24]. Many studies have reported that natural compounds can prevent cognitive deficits caused by scopolamine in mice via conducting behavior tests [3,6]. The aim of this study was to investigate the neuroprotective effects of SM70EE against TMT-induced neurotoxicity in HT-22 neuronal cells. Furthermore, we investigated whether SM70EE can prevent learning and memory impairment caused by scopolamine-induced neurotoxicity in mice.

## 2. Results

### 2.1. The Effects of SM70EE on Cell Viability and Neuronal Death in HT-22 Cells

**Figure 1:**
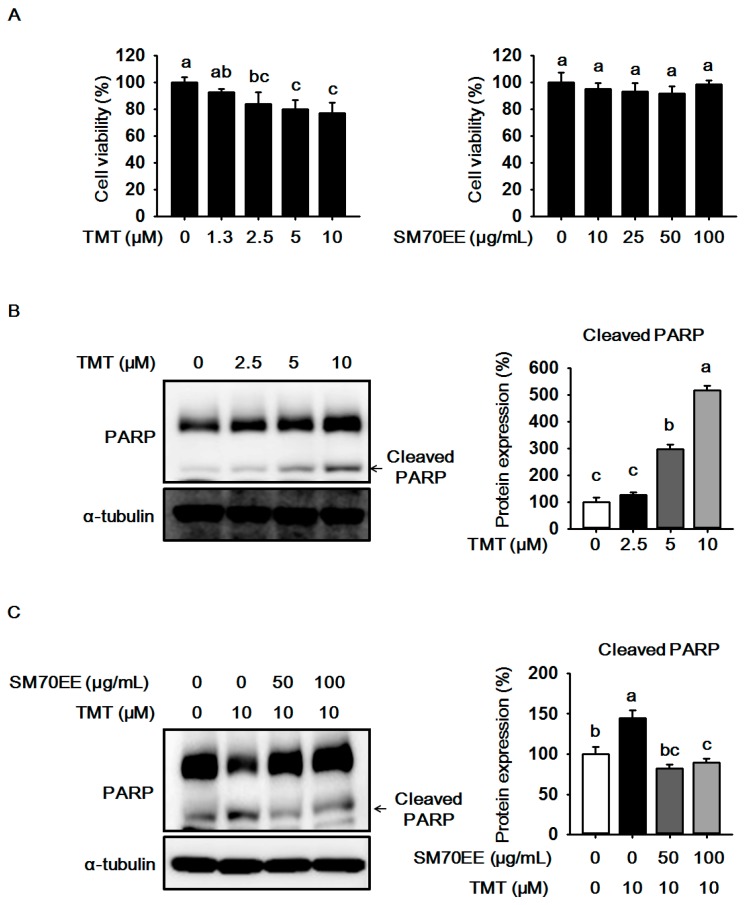
The effects of SM70EE on cell viability and neuronal death in HT-22 cells. (**A**) Cell viability was measured by a MTT assay. The experiments were performed in hexa-plicate; (**B**) HT-22 cells were treated with up to 10 μΜ TMT. Cell lysates were subjected to western blot analysis to analyze to the expression of PARP; (**C**) HT-22 cells were pre-treated 50 and 100 μg/mL SM70EE for 4 h before treatment with 10 μM TMT for 24 h. Cell lysates were subjected to western blot analysis to probe the expression of PARP. The protein expression level was quantified using Image J software and normalized against α-tubulin and control. Results were analyzed by one-way analysis of variance (ANOVA) and Duncan’s multiple range tests. The *p*-value in the multiple comparison results (e.g., a, b, and c) indicate significant differences among the groups (*p* < 0.05).

We performed a MTT assay to investigate the cytotoxicity in HT-22 cells treated with up to 10 μM TMT. As shown in Figure 1A, cell viability was reduced by 10 μM TMT in a dose-dependent manner. We therefore considered that 10 μM TMT is an appropriate concentration which induced neurotoxicity in HT-22 cells. Also, we measured cytotoxicity with up to 100 μg/mL SM70EE to decide its non-toxic concentration in HT-22 cells. At 100 μg/mL SM70EE was found to not be toxic to cells. Therefore, we selected the concentrations of 10 μM TMT, 50, and 100 μg/mL SM70EE for further investigation. 

The expression of PARP cleavage is increased by TMT in neuronal cells [10], so we analyzed the expression of PARP cleavage in HT-22 cells treated with up to 10 μM TMT. As shown in Figure 1C, PARP cleavage was elevated by 10 μM TMT. However, 50 and 100 μg/mL SM70EE inhibited the expression of PARP cleavage, suggesting that SM70EE may prevent neuronal cell death via suppressing PRAP cleavage caused by TMT-induced neurotoxicity in HT-22 cells.

### 2.2. SM70EE Suppresses ROS Production against TMT-Induced Neurotoxicity in HT-22 Cells

**Figure 2:**
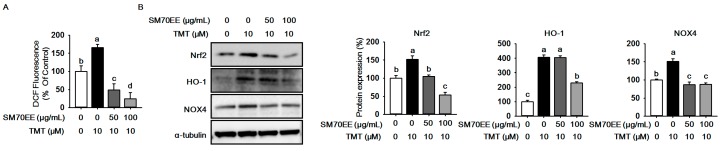
SM70EE inhibits ROS production in TMT-induced neurotoxicity in HT-22 cells. (**A**) HT-22 cells were pretreated 50 and 100 μg/mL SM70EE for 4 h before treatment with 10 μM TMT for 24 h. ROS production was measured by a DCF-DA assay. The experiments were performed in hexa-plicate; (**B**) HT-22 cells were pre-treated 50 and 100 μg/mL SM70EE for 4 h before treatment with 10 μM TMT for 24 h. Cells lysates were subjected to western blot analysis to measure the levels of HO-1, NOX4, and SOD2. The protein expression level was quantified using Image J software and normalized against α-tubulin and control. Results were analyzed by one-way ANOVA and Duncan’s multiple range tests. The *p*-value in the multiple comparison results (e.g., a, b, c, and d) indicate significant differences among the groups (*p* < 0.05).

TMT neurotoxicity augments ROS production and oxidative stress in hippocampal cells [2,11]. To investigate whether SM70EE inhibits ROS generation caused by TMT neurotoxicity, we performed a DCF-DA assay to measure ROS production. As shown Figure 2A, TMT elevated ROS generation, while ROS production was greatly decreased by SM70EE. As shown in Figure 2B, the expressions of Nrf2, HO-1, and NOX4 were elevated by TMT, whereas SM70EE decreased the expressions of Nrf2, HO-1, and NOX4 in HT-22 cells. These data indicate that SM70EE may inhibit ROS generation and protect against TMT neurotoxicity through down-regulating oxidative stress-associated enzymes.

### 2.3. SM70EE Prevents Neuronal Cell Damage Caused in HT-22 Cells by TMT-Induced Neurotoxicity via Enhancing BDNF/CREB Signaling Pathways and Suppressing AChE Activity 

**Figure 3:**
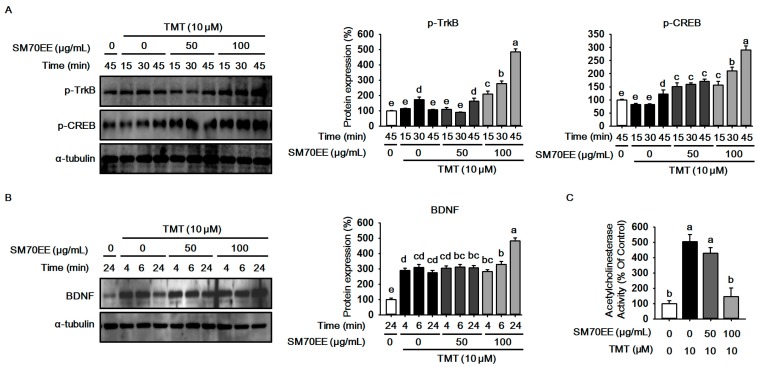
SM70EE prevents neuronal cell damage through enhancing BDNF/CREB signaling pathways and suppressing AChE activity against TMT-induced neurotoxicity in HT-22 cells. (**A**) Proteins were harvested at the indicated time points and subjected to western blot analysis to probe the expressions of p-CREB, p-TrkB; (**B**) Proteins were harvested at the indicated time points and subjected to western blot to analyze to the expression of BDNF. The protein expression level was quantified using Image J software and normalized against α-tubulin and control; (**C**) HT-22 cells were pre-treated 50 and 100 μg/mL SM70EE for 4 h before treatment with 10 μM TMT for 24 h. AChE activity was measured by Ellman method. The experiments were performed in tri-plicate. Results were analyzed by one-way ANOVA and Duncan’s multiple range tests. The *p*-value in the multiple comparison results (e.g., a, b, c, and d) indicate significant differences among the groups (*p* < 0.05).

Neuroprotective factors such as BDNF, CREB, and TrkB are increased to defend and protect cells against TMT-induced neuronal damage [2,15]. To examine whether SM70EE has neuroprotective effects against TMT neurotoxicity, we performed western blot analyses to analyze the expressions of p-TrkB and p-CREB in HT-22 cells. As shown in Figure 3A,B, the expressions of p-TrkB and p-CREB were increased by TMT. SM70EE markedly elevated the expressions of p-TrkB and p-CREB. Besides, the expression of BDNF had the same tendency as p-TrkB and p-CREB. Additionally, the other factor, AChE activity, is increased by TMT neurotoxicity [4]. We analyzed AChE activity to examine whether SM70EE inhibits AChE activity. As shown in Figure 3C, SM70EE suppressed AChE activity. These findings indicate that SM70EE-induced BDNF and AChE changes could partially prevent neurotoxicity induced by TMT in HT-22 cells.

### 2.4. SM70EE Prevents Learning and Memory Impairment against Scopolamine-Induced Neurotoxicity in Mice

**Figure 4:**
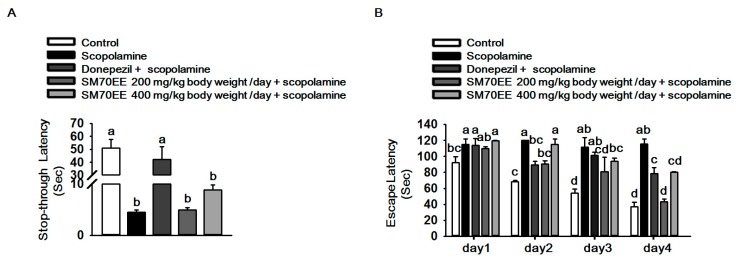
SM70EE prevents learning and memory impairment caused by scopolamine-induced neurotoxicity in mice. (**A**) Mice were subjected to a passive avoidance test; (**B**) Mice were subjected to a Morris water maze test. Results were analyzed by one-way ANOVA and Duncan’s multiple range tests. The *p*-value in the multiple comparison results (e.g., a, b, c, and d) indicate significant differences among the groups (*p* < 0.05).

To determine the effects of SM70EE on cognitive deficits, we performed passive avoidance and Morris water maze tests in mice which were orally administered 200/400 mg/kg body weight/day SM70EE, and 1 mg/kg body weight/day donepezil for 90 min before treatment with 1 mg/kg body weight/day scopolamine. As shown in Figure 4A, the step-through latency time of the scopolamine group (4.0 s) was decreased compared with control group. However, the step-through latency time was slightly increased by 400 mg/kg body weight/day SM70EE (8.8 s). We also measured the escape latency time through performing Morris water maze tests for 4 days. As shown in Figure 4B, the scopolamine treatment group of spent more time to find the platform, but the escape latency time was decreased by SM70EE. These findings indicated that SM70EE would prevent cognitive deficits caused by scopolamine-induced neurotoxicity in mice. To detect C-PC and chlorophyll a, we performed HPLC-PDA analysis. Our data showed that C-PC and chlorophyll a, which are active compounds in *Spirulina maxima* were present in SM70EE (Supplementary Material, Figure S1).

## 3. Discussion

We investigated whether SM70EE prevents mechanisms affected by TMT neurotoxicity such as neuronal cell death, oxidative stress, neurotransmitter dysfunction, and neuroprotective factor levels in HT-22 cells. Furthermore, we examined whether SM70EE prevents memory and learning impairment induced by scopolamine. Especially, we conducted experiments to examine our hypothesis which enhancement of CREB/BENF signaling pathways by SM70EE may contribute to neuroprotection against neurotoxicity.

Neurotoxicity leads to neuronal cell death by affecting several mechanisms. One of the various molecular events increases ROS production via stimulating oxidative stress-associated enzymes such as Nrf2, HO-1, and NOX4, which leads to oxidative stress [11,14]. NOX4 is one of the pro-oxidant enzymes that produce ROS and plays a key role as the electron donor for NADPH [25]. The other enzymes, Nrf2/HO-1, enhance the antioxidant response to TMT neurotoxicity [14]. We observed that SM70EE inhibited ROS production and down-regulated NOX4, Nrf2, and HO-, proving the hypothesis that SM70EE could contribute to the reduction of NOX4, Nrf2, and HO-1 and prevent ROS generation.

Recently, studies have reported that BDNF/CREB signaling pathways stimulate antioxidant response to protect from neuronal cell damage against oxidative stress and prevent learning deficits [26,27,28]. We next analyzed the expressions of p-TrkB, p-CREB, and BDNF. BDNF/CREB signaling pathways which are neuroprotective signaling cascades that promote neuronal survival and protect neuronal cells in the hippocampus from damage. The expressions of p-TrkB, p-CREB, and BDNF were enhanced to protect neuronal cells damaged by neurotoxicity [2,29]. We observed that BDNF/CREB signaling pathways activated to protect cells damaged by TMT, however, the expressions of p-CREB, p-TrkB, and BDNF were markedly increased by SM70EE. We therefore consider that SM70EE might prevent neuronal cell damage via enhancing BDNF/CREB signaling pathways against TMT neurotoxicity.

Also, we measured AChE activity to investigate the relation between cognitive deficits and BDNF/CREB signaling cascades. It has been reported that AChE activity, one of the risk factors in AD, is increased by neurotoxicity in neuronal cells [4,30]. Besides, a study has elucidated that elevation of AChE activity decreases the expression of BDNF [31,32]. Our data showed that SM70EE markedly inhibited AChE activity. We considered that SM70EE would suppress AChE activity via promoting activation of BDNF/CREB neuroprotective signaling pathways. These molecular events by neurotoxicity consequently stimulate PARP cleavage which is an apoptosis-associated protein and results in neuronal cell death [4,10]. We observed that SM70EE inhibited the expression of PARP cleavage. These findings suggested that SM70EE might prevent some mechanisms related with neurotoxicity such as oxidative stress, neurotransmitter dysfunction and neuronal death via stimulating activation of BDNF/CREB signaling pathways in HT-22 cells. 

Taken together the previous studies and our data show that neurotoxicity may be related with cognitive deficits (Figure 3). We next conducted behavior tests using scopolamine in mice. We observed that SM70EE increased step-through latency time and decreased the escape latency time in which mice find the platform. These findings showed that SM70EE may prevent cognitive deficits and protect against scopolamine-induced neurotoxicity in mice.

## 4. Materials and Methods

### 4.1. Preparation of SM70EE

SM70EE was provided by the Korea Institute of Ocean Science & Technology (Jeju, Korea). SM70EE was prepared by a two-step method. In the first step, *S. maxima* was extracted with 70% ethanol with ultrasonic wave assistance at room temperature for about 8 h. Next, the *S. maxima* was extracted at 65–70 °C for about 4 h until the end of the analysis.

### 4.2. Materials

HT-22 cells were provided from Dr. Hyun-Yon Lee (Seowon University, Cheongju, Korea). Dulbecco’s modified Eagle’s medium (DMEM), fetal bovine serum (FBS), penicillin-streptomycin (P/S), and phosphate-bufferedsaline (PBS) were purchased from Gibco (Gaithersburg, MD, USA). Trimethyltin (TMT) was obtained from Sigma (St. Louis, MO, USA). Thiazolyl blue tetrazolium bromide (MTT) was purchased from Alfa Aesar Chemical Inc. (Ward Hill, MA, USA). An Aamplite colorimetric acetylcholinesteraseassay kit was purchased from AAT Bioquest (Sunnyvale, CA, USA).The peroxide-sensitive fluorophore 2,7′-dichlorodihydrofluoresceindiacetate (DCF-DA) was purchased from CELL BIOLABS (San Diego, CA, USA). Unless noted otherwise, all other chemicals were purchased from Sigma (St. Louis, MO, USA). Antibodies specific for PARP, p-CREB, p-TrkB, BDNF, SOD2, NOX4, HO-1, and α-tubulin were obtained from Santa Cruz (Dallas, TX, USA).

### 4.3. Animals

The project was approved by the institutional animal care and use committee (IACUC) of Kangwon National University. ICR mice (male, 3 weeks old) were obtained from the Dae Han Biolink Co. (Eumseong, Korea). The room was maintained with a 12 h light-dark cycle at 20 ± 3 °C. After adaptive period for a week, the mice were divided into five groups (*n* = 6 per group). The mice were fed with chow diet and orally administered by gavage once a day for 3 weeks (control, 200 and 400 mg/kg body weight/day SM70EE, and 1 mg/kg body weight/day donepezil). The mouse group of SM70EE and donepezil were administered orally 90 min before treatment with 1 mg/kg body weight/day scopolamine. The SM70EE was dissolved in 0.5%carboxymethylcellulose. The test was performed 30 min after scopolamine treatment.

### 4.4. Passive Avoidance Test

The mice were tested in a passive avoidance shuttle box (Gemini, San Diego, CA, USA), which was divided into two chambers (illuminated and dark). The first day, mice were performed a training trial in the shuttle box. Each mouse was placed in the illuminated compartment for 2 min, and then when the mouse entered the dark compartment the door was closed, mouse feet received an electric shock (0.5 mA, 2 s). After 24 h, the test was given again, the mouse was placed in the illuminated compartment and the latency time to enter the dark compartment was measured (maximum testing time of the step-through latency was 180 s) [33].

### 4.5. Morris Water Maze Test

The water maze consisted of a circular pool (90 cm in diameter) filled with water up to 30 cm and maintained at 20 ± 1 °C and area of the maze were defined as four equal quadrants. A white escape platform was placed in the center of one quadrant and submerged 1 cm below the surface of the water. All swimming behaviors of the mice were monitored and analyzed by a Smartver.2.5.21 (Panlab, Cornellà, Spain) video-tracking system. Mice were analyzed 3 times per day for 4 days. The time required to find the platform was investigated to determine the memory of the mice (maximum testing time of the escape latency was 120 s) [34].

### 4.6. Cell Culture 

HT-22 cells were grown in DMEM medium containing 3.7 g/L sodium bicarbonate, 1% P/S and 10% FBS at 37 °C in 5% CO_2_. When the cells were used in the experiments, the medium was replaced with serum free media overnight. Cells were pre-treated 50 and 100 μg/mL SM70EE for 4 h before treatment with 10 μM TMT for the indicated time. TMT and SM70EE were dissolved in deuterium-depleted water.

### 4.7. MTT Assay

HT-22 cells (1 × 10^4^ cells/well) were seeded in 96 well plates. Cells were pre-treated 50 and 100 μg/mL SM70EE for 4 h before treatment with 10 μM TMT for 24 h. The treated cells were stained with MTT for 3 h at 37 °C in 5% CO_2_. MTT formazan was quantified by using DMSO and was determined by a Powerwave HT ELISA reader (BioTek, Winooski, VT, USA) at 580 nm absorbance.

### 4.8. Measurement of Intracellular ROS Production

HT-22 cells (1 × 10^4^ cells/well) were seeded in 96 well plates. Cells were pre-treated 50 and 100 μg/mL SM70EE for 4 h before treatment with 10 μM TMT for 24 h. Then, 20 μM DCF-DA was added in treated cells. After incubation for 30 min at 37 °C in 5% CO_2_, absorbance was determined by ELISA reader Powerwave HT (BioTek) at 480 to 530 nm.

### 4.9. Acetylcholinesterase Activity

HT-22 cells (1 × 10^4^ cells/well) were seeded in 96 well plates. Cells were pretreated with 50 and 100 μg/mL SM70EE for 4 h before treatment with 10 μΜ TMT for 24 h. Then, 50 μL AChE reaction mixtures were added. After, incubation for 10 min at 37 °C in 5% CO_2_, absorbance was determined on a Powerwave HT ELISA reader (Bio Tek) at 410 nm.

### 4.10. Western Blotting

Cells were harvested by lysis buffer containing protease inhibitors and phosphatase inhibitor cocktail II, III. Protein extracts (25 μg) were separated via SDS-PAGE and transferred to a PVDF membrane. The membranes were blocked with 5%skim milk and immunoblotted with primary antibodies specific for indicated proteins for overnight. Secondary antibodies conjugated with horseradish peroxidase (1:5000) were applied for 4 h. The bands were visualized by enhanced chemiluminescence, and proteins were detected with LAS image software (Fuji, Valhalla, NY, USA). The protein expression level was quantified using Image J software and normalized against α-tubulin and control.

### 4.11. Statistical Analyses

All statistical analyses were performed by using the Statistical Package for Social Sciences version 12.0 (SPSS Inc., Armonk, NY, USA). A one-way analysis of variance (ANOVA) was used for comparisons among the groups. Significant differences between the mean values were assessed using Duncan’s test. The *p*-value in the multiple comparison results (e.g., a, b, c, and d) indicate significant differences among the groups (*p* <0.05). 

## 5. Conclusions

Our results suggested that SM70EE might partially inhibit oxidative stress induced by TMT in HT-22 cells. Notably, we considered that SM70EE would partially prevent neurotoxicity of TMT via promoting activation of BDNF/CREB signaling pathways. In addition, our data indicated that SM70EE may prevent the cognitive deficits caused by scopolamine-induced neurotoxicity in mice. We suggested that SM70EE might be a potential natural compound against neurotoxicity of anti-cholinergic drugs.

## Supplementary Materials

Supplementary materials are available online. Figure S1: HPLC chromatogram of C-PC and chlorophyll a in SM70EE.
