# Supplementary material for: Spirulina maxima Extract Prevents Neurotoxicity via Promoting Activation of BDNF/CREB Signaling Pathways in Neuronal Cells and Mice
Source: Molecules. 2017 Aug 17;22(8):1363. doi: 10.3390/molecules22081363 (PMC6151979; doi:10.3390/molecules22081363)
Supplement: Supplementary file 1 [file molecules-22-01363-s001.pdf]

Supplementary Material

SpirulinaMaxima Extract Prevents Neurotoxicity via Promoting Activation of BDNF/CREB Signaling Pathways in Neuronal Cells and Mice

Eun-Jeong Koh [1,†], Young-Jin Seo [1,†],Jia Choi [1], Hyeon Yong Lee [2], Do-Hyung Kang [3], Kui-Jin Kim [1,*]and Boo-Yong Lee [1,*]

Materials and Methods

C-PC high performance liquid chromatography (HPLC) analysis

The presence of C-PC in SM70EE was assayed by HPLC (Agilent 1260 series, Agilent, Santa Clara, CA, USA) with photodiode array (PDA) detection using a Sepax Bio-C4 column (4.6 × 150 mm, 3 μm, Sepax Technologies, Inc., Newark, DE, USA). The elution was performed using a linear gradient from 50 to 100 % (*v*/*v*) aqueous acetonitrile (ACN). The injection volume was 20 μL. The PDA detector was set at 620 nm for C-PC.

Chlorophyll a HPLC analysis

The constituent chlorophyll a from SM70EE was detected by HPLC with PDA detection using a Jupiter 300 C18 column (250 × 4.6 mm, 5 μm, Phenomenex, Torrance, CA, USA). The mobile phase was a mixture of solvent A (acetonitrile), solvent B (methanol), and solvent C (ammonium acetate). The elution started using an (A:B:C = 4:2:4) solvent mixture for 5 min. After 5 min, the mixture solvent ratio was changed (A:B:C = 6:2:2) for 15 min. Lastly, the mobile phase composition was changed back to the first step composition until the end of the analysis. The injection volume was 20 μL. The PDA detector was set at 420 nm which is appropriate for chlorophyll a.

A

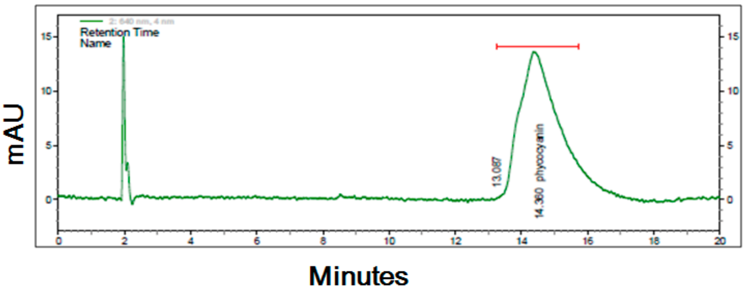

B

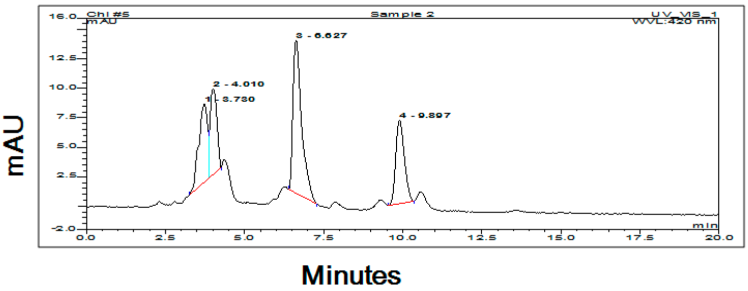

C

	C-phyocyanin	Chlorophyll a
Retention time (min)	14.23	6.61
Concentration (mg/mL)	0.107	0.054
Concerntraion %	10.7 %	5.4 %

Supplementary Figure 1. HPLC chromatograms of C-PC and chlorophyll a in SM70EE**.** (**A**) C-PC from SM70EE analyzed by HPLC-PDA. (**B**) Chlorophyll a from SM70EE measured by HPLC-PDA. (**C**) Retention time and quantification of C-PC and chlorophyll a.
